# Modulating Beta-Cardiac Myosin Function at the Molecular and Tissue Levels

**DOI:** 10.3389/fphys.2016.00659

**Published:** 2017-01-09

**Authors:** Wanjian Tang, Cheavar A. Blair, Shane D. Walton, András Málnási-Csizmadia, Kenneth S. Campbell, Christopher M. Yengo

**Affiliations:** ^1^Department of Cellular and Molecular Physiology, Pennsylvania State University College of MedicineHershey, PA, USA; ^2^Department of Physiology, University of KentuckyLexington, KY, USA; ^3^Department of Biochemistry, Eötvös Loránd UniversityBudapest, Hungary; ^4^Division of Cardiovascular Medicine, University of KentuckyLexington, KY, USA

**Keywords:** myosin, actin, muscle contraction, molecular motors, cardiomyopathy

## Abstract

Inherited cardiomyopathies are a common form of heart disease that are caused by mutations in sarcomeric proteins with beta cardiac myosin (*MYH7*) being one of the most frequently affected genes. Since the discovery of the first cardiomyopathy associated mutation in beta-cardiac myosin, a major goal has been to correlate the *in vitro* myosin motor properties with the contractile performance of cardiac muscle. There has been substantial progress in developing assays to measure the force and velocity properties of purified cardiac muscle myosin but it is still challenging to correlate results from molecular and tissue-level experiments. Mutations that cause hypertrophic cardiomyopathy are more common than mutations that lead to dilated cardiomyopathy and are also often associated with increased isometric force and hyper-contractility. Therefore, the development of drugs designed to decrease isometric force by reducing the duty ratio (the proportion of time myosin spends bound to actin during its ATPase cycle) has been proposed for the treatment of hypertrophic cardiomyopathy. Para-Nitroblebbistatin is a small molecule drug proposed to decrease the duty ratio of class II myosins. We examined the impact of this drug on human beta cardiac myosin using purified myosin motor assays and studies of permeabilized muscle fiber mechanics. We find that with purified human beta-cardiac myosin para-Nitroblebbistatin slows actin-activated ATPase and *in vitro* motility without altering the ADP release rate constant. In permeabilized human myocardium, para-Nitroblebbistatin reduces isometric force, power, and calcium sensitivity while not changing shortening velocity or the rate of force development (*k*_tr_). Therefore, designing a drug that reduces the myosin duty ratio by inhibiting strong attachment to actin while not changing detachment can cause a reduction in force without changing shortening velocity or relaxation.

## Introduction

Inherited cardiomyopathies caused by mutations in sarcomere protein-coding genes are a significant cause of cardiovascular diseases in people of all ages (Morimoto, 2007; Watkins et al., 2011). Hypertrophic cardiomyopathy (HCM) is the most common form of inherited cardiomyopathy, and the primary cause of sudden cardiac death in young adults (Maron, 2004; Efthimiadis et al., 2014; Maron et al., 2014). The latest revised HCM prevalence is about 1 in 200 of the general population including mutation carriers at risk for developing a phenotype (Semsarian et al., 2015). HCM manifests as left ventricle hypertrophy featuring cardiomyocyte disarray and fibrosis, a thickening of the left ventricular wall and decreased coronary artery blood flow during diastole (Maron, 2002; Maron et al., 2006; Watkins et al., 2011; Vakrou and Abraham, 2014). Dilated cardiomyopathy (DCM) has an estimated prevalence of 1 in 2500 individuals, and the cases with a genetics etiology account for ~50% (Taylor et al., 2006; Towbin, 2014). DCM is characterized by the thinning of one or both ventricular walls, an enlarged left ventricular chamber, and insufficient systolic contraction (Luk et al., 2009; Hershberger et al., 2010; McNally et al., 2013). Restrictive, arrhythmogenic right ventricular, left ventricular non-compaction, and other types of cardiomyopathies have been classified as well, but are less prevalent in the general population (Elliott et al., 2008; Watkins et al., 2011; Towbin, 2014).

Cardiomyopathy mutations are commonly found in the myosin heavy chain 7 gene (*MYH7*) encoding human β-cardiac myosin heavy chain (M2β) (Xu et al., 2010), which is the motor that drives contraction of the ventricular myocardium. Single point mutations in M2β are capable of disrupting motor function. Identification of disease mutations has raised expectations for disease prediction and novel therapeutic strategies. More than 300 pathogenic mutations in M2β are distributed throughout the whole myosin molecule, and there is no consensus about the detailed mechanisms behind the impact of these mutations (Moore et al., 2012). The mechanisms responsible for altering motor functions are varied, and are likely dependent on the locations of the mutation (Moore et al., 2012; Colegrave and Peckham, 2014; Homburger et al., 2016).

### Myosin structure-function

**Figure 1:**
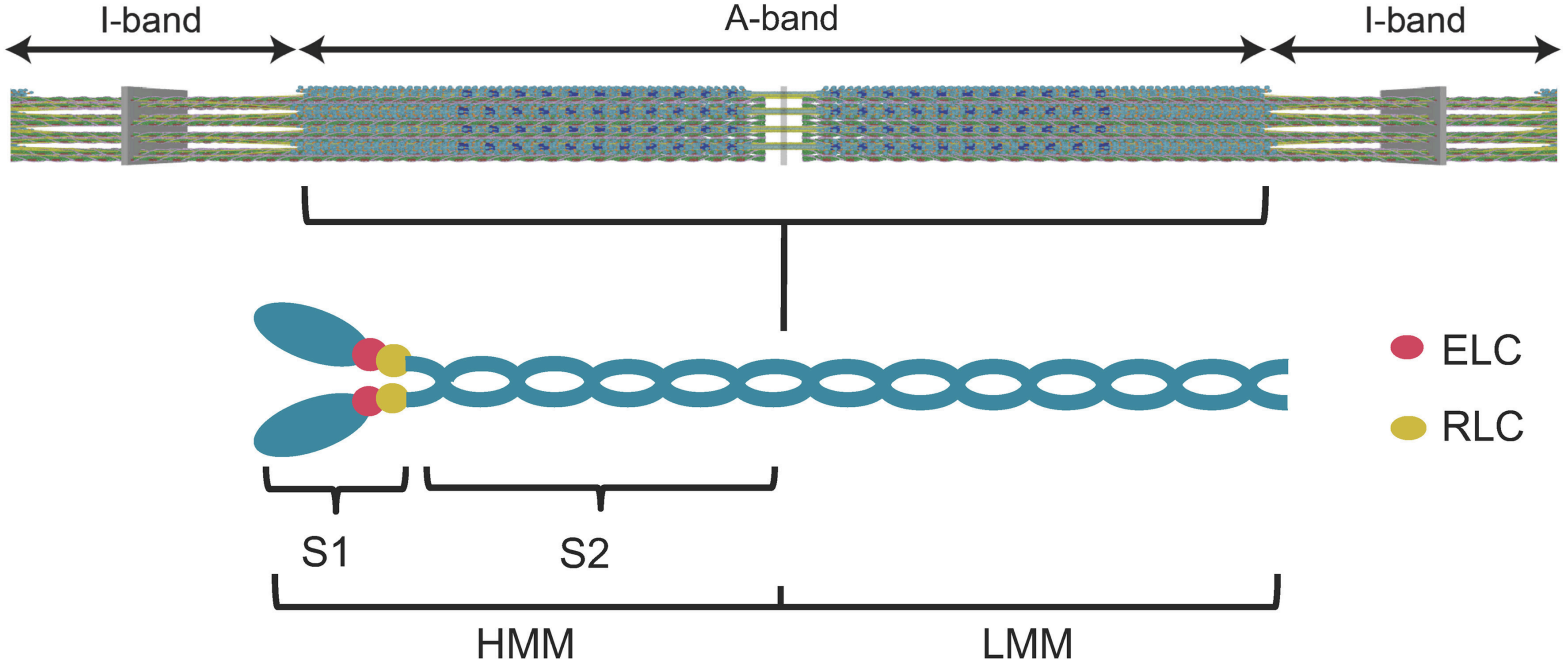
**Diagram of the muscle sarcomere and the myosin molecule**. A simplified diagram of the sarcomere **(top panel)** demonstrates the location of the myosin thick filaments (A-band) and the actin thin filaments (I-band). A diagram of the myosin molecule **(lower panel)** demonstrates its overall structure. The myosin heavy chains, light chains (essential light chain, ELC, regulatory light chain, RLC), subfragment 1 (S1) (utilized in the current study), subfragment 2 (S2), heavy meromyosin (HMM), and light meromyosin (LMM) are labeled.

Many years of research has established that myosin is the motor protein that converts chemical energy into mechanical work and drives the shortening of muscle and other forms of actomyosin-based force generation. The main components of the muscle sarcomere are thick and thin filaments. The thick filaments are composed of myosin molecules that form cross-bridges that interact with thin filaments composed of actin. Myosin consists of two heavy chains, each with two associated light chains, an N-terminal motor domain, and C-terminal coiled-coil tail that allows dimerization and incorporation into the thick filaments (Figure 1). There are several proteins associated with the thick filaments (e.g., myosin binding protein C, Titin) involved in contractile regulation. Thin filaments contain actin and regulatory proteins (tropomyosin and troponin complex), which are important for mediating the Ca^2+^-induced activation of the thin filaments.

### Myosin ATPase cycle

**Figure 2:**
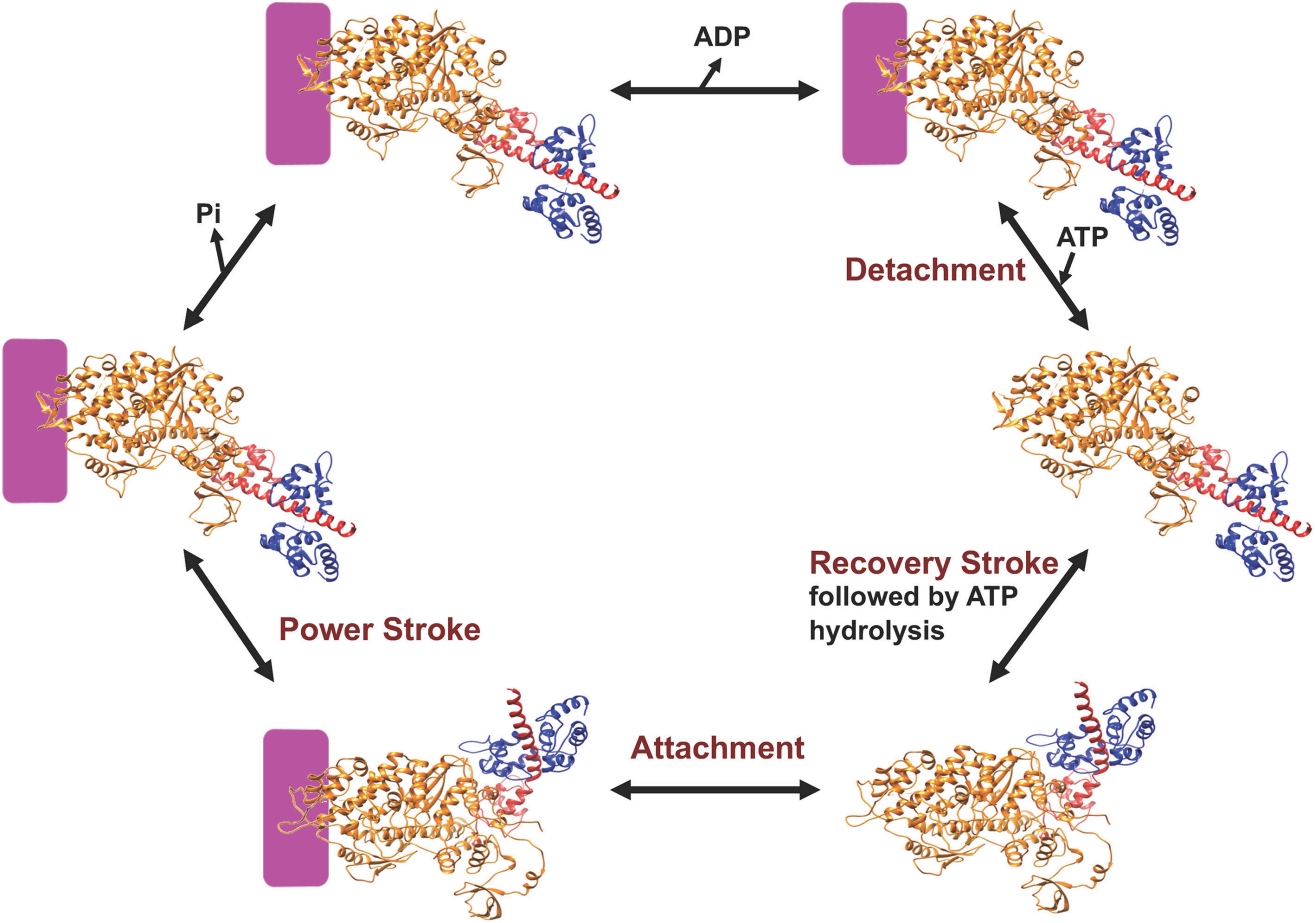
**Diagram of the myosin ATPase cycle**. A simplified model of the myosin ATPase cycle shown with two structural states, the pre-power stroke and post-power stroke states. The pre-power stroke state is represented by the smooth muscle myosin structure in the presence of ADP.ALF4 (PDB ID: 1BR4) and the post-power stroke state is represented by the APO myosin V structure (PDB ID: 1OE9). The lever arm is shown in red and the light chain in blue. Actin is indicated by the purple box and the individual steps in the ATPase cycle are described in the text.

Myosin is an ATP-dependent molecular motor that cyclically interacts with actin filaments with weak and strong actin-binding states. Figure 2 describes the key steps in the catalytic cycle and the proposed structural changes that occur in each step. ATP binding to myosin causes a conformational change in the actin binding region resulting in weak actin affinity, and formation of the pre-power stroke state of the lever arm (recovery stroke). ATP is hydrolyzed while myosin is dissociated from actin in a weak actin-binding state. Myosin binding to actin with the hydrolyzed products accelerates the release of phosphate and then ADP, which results in force generation (power stroke). Recent work in the Yengo lab on myosin V has shown that the lever arm swing occurs in two steps, a fast step that gates phosphate release and a slow step coupled to ADP release (Trivedi et al., 2015). Studies with skeletal muscle myosin also demonstrate a rapid movement of the lever arm prior to phosphate release (Muretta et al., 2015). Alternatively, evidence from x-ray crystallography suggests that the movement of phosphate from the active site into the phosphate release tunnel is required for the movement of the lever arm, while release of phosphate from the tunnel into solution occurs after the lever arm swing (Houdusse and Sweeney, 2016). Muscle fiber studies have provided evidence that phosphate release occurs after force generation (Dantzig et al., 1992) or is orthogonal to the power stroke (Caremani et al., 2013, 2015), while correlating the biochemical, structural, and muscle fiber experiments remains a challenge.

### Force-velocity properties of muscle

The chemomechanical ATPase cycle contains two fundamental parts, weak actin-binding states (M.ATP and M.ADP.Pi) and strong actin-binding states (A.M.ADP.Pi, AM.ADP and A.M). Force generation occurs in the strong binding states, during which the myosin power stroke generates a displacement (step size = 5–10 nm in muscle myosins) of the actin filament (Spudich, 2014). The duty ratio is the fraction of ATPase cycle time myosin is in the strong binding states, which determines the number of strongly bound myosin heads interacting with the thin filaments at any time. Each myosin head is an independent motor and produces its own intrinsic force (f). The overall isometric force (F) is the intrinsic force (f) multiplied by the number of force-generating myosin heads, which can be expressed as the following equation (Spudich, 2014):

$$\begin{array}{l} \text{F}=\text{f }\times \;{\text{N}}_{\text{total}}\;\times \text{ duty ratio} \end{array}$$

where N_total_ is the number of heads that are potentially able to bind to the thin filaments. The maximum shortening velocity is thought to depend on the myosin step size (unitary displacement—d_uni_) and the period of time myosin is attached to actin (t_on_). Thus, the following equation is often used to describe the maximum shortening velocity (Warshaw, 2004):

$$\begin{array}{l} {\text{V}}_{\text{max}}\;=\;{\text{d}}_{\text{uni}}/{\text{t}}_{\text{on}} \end{array}$$

Since these parameters can be measured with isolated myosin, it is possible to correlate the individual properties of myosin with the contraction parameters in muscle. However, the t_on_ is altered by the presence of load, as established in muscle fiber studies (Piazzesi et al., 2002; Reconditi et al., 2004) and further explored in single molecule mechanics studies (Sung et al., 2015; Greenberg et al., 2016). In addition, the factors that limit V_max_ are controversial with some studies demonstrating detachment rate (1/t_on_) correlates well with V_max_ (Siemankowski et al., 1985; Nyitrai et al., 2006; Yengo et al., 2012) and other studies demonstrating attachment rate limits V_max_ (Haldeman et al., 2014; Brizendine et al., 2015).

The *in vitro* motility assay is commonly used to examine the force generating properties of purified myosin (Kron et al., 1991). In this assay myosin is adhered to a microscope cover slip and the sliding velocity of fluorescently labeled actin is monitored in the presence of ATP. The sliding velocity generated by an ensemble of myosin motors is thought to correlate to the shortening velocity measured in muscle (Howard, 2001). In order to examine d_uni_, f, and t_on_, the single molecule laser trap motility assay is often used (Simmons and Finer, 1994; Sivaramakrishnan et al., 2009). In this assay a single actin filament is strung between two beads that are each trapped with laser tweezers and when a single myosin molecule is brought close to the actin filament individual displacements (d_uni_) are measured. The single molecule laser trap studies are typically performed at low ATP concentrations which can create uncertainty in determining t_on_ and correlating it with muscle fiber studies (Tyska and Warshaw, 2002). The stiffness of the laser trap can allow determination of the force generated by a single myosin head (f), but due to the large compliance of the laser trap the force can be underestimated (Spudich et al., 2011).

### The impact of mutations in human β-cardiac myosin

Humans predominantly express the slow β-cardiac myosin isoform in ventricles but most studies examining the impact of mutations have been performed in mice which express α-cardiac myosin, a faster cardiac myosin isoform (Deacon et al., 2012). This has complicated the interpretation of the experimental data because mutations in α-cardiac myosin have different effects than mutations in β-cardiac myosin (Lowey et al., 2008; Palmer et al., 2008; Witjas-Paalberends et al., 2014; Nag et al., 2015). Other studies have examined human muscle fibers purified from skeletal muscle biopsies or from ventricular samples obtained from patients who had cardiac surgeries (Köhler et al., 2002; Seebohm et al., 2009; Brenner et al., 2012; Kraft et al., 2013; Witjas-Paalberends et al., 2014). Measurements on human recombinant β-cardiac myosin are just beginning to be reported and are promising for examining large numbers of different mutations to establish structure-function relationships. Recent studies have demonstrated that some mutations have a relatively small impact on the key parameters mentioned above (f, V, t_on_, d_uni_) (Alpert et al., 2005; Moore et al., 2012; Nag et al., 2015). Thus, it is still unclear how the point mutations lead to impaired cardiac muscle function and hypertrophy.

### Current treatments

Despite the lack of a clear understanding of the molecular mechanisms of cardiomyopathies, symptom-based inotropic drugs are still the conventional clinical pharmacological therapy (Maron, 2002; Spirito and Autore, 2006; Vakrou and Abraham, 2014; Tardiff et al., 2015). β-adrenergic antagonists (e.g., Metoprolol and Nebivolol), Ca^2+^ channel blockers (e.g., Verapamil and Diltiazem), Na^+^ channel blockers (e.g., Disopyramide), antiarrhythmic agents (e.g., Amiodarone), and angiotensin II receptor antagonists (e.g., Losartan) are currently used in the clinic to alleviate the symptoms of HCM (Vakrou and Abraham, 2014; Tardiff et al., 2015). For DCM patients, angiotensin-converting enzyme inhibitors, β-adrenergic blockers, aldosterone inhibitors, and angiotensin receptor blockers have been used clinically (Elliott, 2000; Taylor et al., 2006; Luk et al., 2009). An implantable cardioverter-defibrillator has been shown as the only effective way to prevent sudden cardiac death, and heart transplantations are usually needed for cardiomyopathy patients with end-state heart failure (Elliott and McKenna, 2004; Efthimiadis et al., 2014).

Additionally, inotropic drugs which directly target sarcomeric proteins are under investigation (Malik et al., 2011; Tardiff et al., 2015; Green et al., 2016). The thin filament has been suggested as an ideal target site to treat cardiomyopathies via altering Ca^2+^ sensitivity. Thick filaments are also being pursued as drug targets. By changing the kinetics of individual steps in the myosin ATPase cycle, small molecule drugs are proposed to change the duty ratio and thus the number of forge-generating myosin heads capable of interacting with the thin filaments. Utilization of drugs that directly target contractile proteins in cardiac muscle is still in its early stages and will require detailed pre-clinical studies that can examine their specific mechanisms of action and off-target effects.

Blebbistatin (Bleb) is a well-established inhibitor of class II myosins and understanding its mechanism of action has been an important step in developing novel inhibitors of myosin based force generation. Bleb was first identified as a muscle and non-muscle myosin II specific inhibitor with a mechanism of binding to the ATPase intermediate with ADP and phosphate and slowing down phosphate release by trapping myosin in a weak actin-binding conformation (Straight et al., 2003; Kovács et al., 2004; Ramamurthy et al., 2004; Farman et al., 2008). Additionally, Bleb has been shown to inhibit striated and smooth muscle myosins but with no effect on unconventional class I, V, and X myosins (Limouze et al., 2004; Dou et al., 2007; Eddinger et al., 2007). Studies of Bleb in rodent cardiac muscle found that Bleb decreased the twitch force of isolated cardiac trabeculae and the shortening velocity of cardiac myocytes in a dose-dependent manner (Dou et al., 2007; Farman et al., 2008). Since Bleb binds near the actin binding region and traps the myosin heads in a weak actin affinity state, it is also proposed to reduce the myosin binding-induced activation of the thin filaments (Ramamurthy et al., 2004; Allingham et al., 2005; Dou et al., 2007). Bleb has also been found to stabilize the helical ordering of myosin heads, a conformation in which myosin heads interact with each other but not with actin (Zhao et al., 2008; Xu et al., 2009). This state has been referred to as the super relaxed state (SRX) and Bleb has been shown to stabilize the SRX by unknown mechanisms (Wilson et al., 2014). The use of Bleb was hindered by its blue light sensitivity, phototoxicity, and poor solubility (Sakamoto et al., 2005; Mikulich et al., 2012), but this has been addressed by the discoveries of highly soluble, non-phototoxic Bleb derivatives [para-Nitroblebbistatin (pN-Bleb), and amino-blebbistatin; Képiró et al., 2014; Várkuti et al., 2016].

In the current study we examined the impact of pN-Bleb on human β-cardiac myosin in both expressed/purified myosin *in vitro* motor assays and in human myocardium fiber mechanics studies. We hypothesized that the less phototoxic pN-Bleb would be able to inhibit the *in vitro* motor properties of human cardiac myosin at the molecular and tissue levels. We proposed that investigating the impact of this drug on human myocardium would lead to insight into strategies for designing cardiac myosin specific drugs. Our results provide evidence of the mechanism of action of pN-Bleb on human β-cardiac myosin and suggest important considerations in designing novel drugs that impact the force and shortening velocity properties of cardiac muscle.

## Methods

### Reagents

ATP was prepared from powder (De La Cruz and Ostap, 2009). 2′-deoxy-ADP labeled with N-Methylanthraniloy at the 3′-ribose position (*mant*ADP) was purchased from Jenna Biosciences. pN-Bleb was obtained from András Málnási-Csizmadia and dissolved in DMSO. All motility experiments with M2β were performed in motility buffer with pCa value of 4.5 (7 mM EGTA, 20 mM Imidazole, 51 mM KCl, 7 mM CaCl_2_, 5.22 mM MgCl_2_, pH 7.0) and other experiments were performed in MOPS 20 buffer (10 mM MOPS, 20 mM KCl, 1 mM MgCl_2_, 1 mM EGTA, 1 mM DTT, pH 7.0). The final concentrations of pN-Bleb are described for each experiment and the final concentration of DMSO was 1% for the motility, ATPase, and ADP release experiments. Further details about the solutions for the muscle mechanics studies are given below. All concentrations listed are final unless stated otherwise.

### Construction of expression plasmids

The human cardiac myosin cDNA (AAA5187.1) was purchased from Thermo Scientific. PCR amplification was used to subclone the M2β subfragment 1 (M2β-S1) construct (amino acids 1–843) into the pshuttle vector (a gift from Dr. Don Winkelmann). M2β-S1 was engineered to contain an N-terminal FLAG tag sequence and C-terminal Avi tag sequence.

### Recombinant adenovirus based expression and purification of M2β-S1 in C_2_C_12_ cells

The production of high titer adenovirus was performed by a method developed in the Winkelmann laboratory (Srikakulam and Winkelmann, 2004; Winkelmann et al., 2015). Homologous recombination was used to produce pAdEasy recombinant adenovirus DNA (pAd.M2β-S1) by transforming the pshuttle.M2β-S1 into *E. coli* BJ5183 cells. The pAd.M2β -S1 was transformed into XL-10 Gold cells for amplification and the pAd.M2β-S1 DNA was digested with Pac1 and transfected into Ad293 cells to allow for virus packaging and amplification. The Ad293 cells were grown in DMEM media supplemented with 10% fetal bovine serum. The large scale virus preparation was performed by infecting 60 plates (145 mm diameter). The virus was harvested with freeze thaw cycles followed by CsCl density sedimentation. The final virus titers were typically 10^10^–10^11^ plaque forming units (PFU) per ml.

C_2_C_12_ cells grown to 90% confluence in DMEM supplemented with 10% fetal bovine serum (typically 20–30, 145 mm diameter plates) were differentiated by changing the media to DMEM supplemented with 10% horse serum and 1% fetal bovine serum. The C_2_C_12_ cells were infected with recombinant adenovirus (5 × 10^8^ PFU/ml) diluted into differentiation media. The media was changed after 2 days and cells were harvested on day 7. The cells were lysed with a 50 ml dounce in lysis buffer (50 mM Tris, pH 7.0, 200 mM KCl, 2 mM ATP, 1 mM ATP, 0.5% Tween20, 0.01 mg/ml aprotenin, 0.01 mg/ml leupetin, 1 mM PMSF) and spun 2 × 15 min at 25 K in a Ti50 rotor at 4°C. The supernatant was added to a 1 ml anti-FLAG M2 resin column, washed with wash buffer (10 mM Tris, pH 7.5, 200 mM KCl, 1 mM EGTA, 1 mM EDTA, 2 mM MgCl_2_, 2 mM ATP, 1 mM DTT, 0.01 mg/ml aprotenin, 0.01 mg/ml leupetin, 1 mM PMSF), and eluted with wash buffer containing FLAG peptide (0.167 mg/ml). The eluted M2β-S1 was subsequently ammonium sulfate precipitated and dialyzed into MOPS 20 buffer overnight at 4°C. M2β-S1 was biotinylated for *in vitro* motility studies by incubating M2β-S1 with BirA (10 μg/ml) for 1 h at 25–30°C, and subsequently ammonium sulfate precipitated and dialyzed into MOPS 20 buffer overnight at 4°C (Lin et al., 2005).

M2β-S1 purity was assessed by coomassie stained SDS-polyacrylamide gels and protein concentration was determined by Bradford assay using BSA as a standard. Similar results were obtained by measuring the absorbance and using the predicted extinction coefficient (ε_280_ = 1.38 × 10^5^ M^−1^·cm^−1^). Skeletal muscle heavy meromyosin (Sk HMM) was prepared from rabbit psoas muscle as described (Swenson et al., 2014). Actin was purified from rabbit skeletal muscle using an acetone powder method (Pardee and Spudich, 1982). The actin concentration was determined by absorbance at 290 nm (ε_290_ = 2.66 × 10^4^ M^−1^·cm^−1^). A molar equivalent of phalloidin was added to stabilize F-actin.

### *In vitro* motility assay

We performed *in vitro* motility assays (Kron et al., 1991) using the recombinantly expressed/purified M2β-S1 and purified Sk HMM. The M2β-S1 experiments were performed in conditions (buffer and temperature) that were similar to the muscle mechanic studies described below. The actin filament sliding assay was performed as previously described (Trivedi et al., 2013; Swenson et al., 2014) except for the method of adhering the myosin to the surface in the case of M2β-S1. Microscope cover slips were coated with 1% nitrocellulose in amyl acetate (Ladd Research). The surface was coated with streptavidin (0.1 mg/ml) and blocked with BSA (1 mg/ml) before the addition of biotinylated M2β-S1 (loading concentration was 0.48 μM). Unlabeled sheared actin (2 μM) followed by an ATP (2 mM) wash was used to prevent interactions with dead heads. Actin labeled with ALEXA (GFP filter; excitation/emission: 500/535 nm) was visualized by fluorescence microscopy. An activation buffer with 1% DMSO or pN-Bleb (0.1, 1, 5, 10, 20, 50 μM) was added to the flow cell to initiate motility. Activation buffer contained the following: 0.35% methylcellulose, 2.5 mM phosphoenolpyruvate, 20 units·ml^−1^ pyruvate kinase, 0.1 mg·ml^−1^ glucose oxidase, 5 mg·ml^−1^ glucose, 0.018 mg·ml^−1^ catalase, and 4.8 mM ATP. The slide was promptly viewed using a NIKON TE2000 microscope equipped with a 60 × /1.4 NA phase objective and a Perfect Focus System. Images were acquired at intervals (appropriate for each condition) for periods of time (3–15 min) using a shutter controlled Coolsnap HQ2 cooled CCD digital camera (Photometrics) binned 2 × 2. Temperature was maintained at 22–24°C and monitored using a thermocouple meter (Stable Systems International). Image stacks were transferred to ImageJ for analysis via MTrackJ (Meijering et al., 2012). The average velocity was determined by tracking actin filaments manually for each condition using ImageJ.

### *In vitro* steady-state ATPase activity

Steady-state ATP hydrolysis by M2β-S1 or Sk HMM (100 nM) in the presence of actin (40 μM) was examined by using the nicotinamide adenine dinucleotide (NADH)-linked assay (De La Cruz et al., 2000; Dosé et al., 2007, 2008; Quintero et al., 2010) in MOPS 20 Buffer with a final MgATP concentration of 1 mM. The assay was performed in an Applied Photophysics stopped-flow (Surrey, UK) in which the NADH absorbance at 340 nm was monitored continuously for 200 s. The data at each actin concentration represents an average of 2 protein preparations.

### Determination of IC50

We plotted the relative ATPase or sliding velocity data as a function of pN-Bleb concentration which allowed us to determine the IC50 by fitting the data to the following equation: Relative activity = 1/{(1+[pN-Bleb]/IC50)}.

### Transient kinetic measurements of ADP-release

We examined the ADP release rate constant of M2β-S1 in the presence of actin. A complex of M2β-S1, actin, and *mant*ADP (0.375, 1, and 10 μM, respectively) was mixed with saturating ATP (1 mM) and the *mant* fluorescence (excitation 290 nm/emission 395 nm long pass filter) was monitored in the stopped-flow. The fluorescence transients were fit with custom software provided with the instrument or Graphpad Prism.

### Human tissue

Myocardial samples were obtained at the University of Kentucky from patients who had end-stage heart failure using the protocol described by Blair et al. (2016). Briefly, through-wall sections of the distal anterior region of the left ventricle were obtained from explanted hearts and dissected transmurally (sub-epicardial, mid-myocardial, sub-endocardial). The experiments described in this manuscript were performed using a total of 24 sub-endocardial samples from 4 patients. All procedures were approved by the University of Kentucky Institutional Review Board and patients gave informed consent.

### *In situ* preparations and experimental set-up

Permeabilized multicellular preparations were obtained using the mechanical digest protocol described by Haynes et al. (2014). Multicellular preparations with a mean length 1047 ± 232 μm were attached between a force transducer (resonant frequency, 600 Hz; model 403, Aurora Scientific, Aurora, Ontario, Canada) and a motor (step time 0.6 ms; model 312B, Aurora Scientific) and stretched to a sarcomere length of 2.24 μm in a solution with a pCa (=−log_10_[Ca^2+^]) of 9.0. The cross-sectional area was 5.07 ± 2.47 × 10^−8^ m^2^ (estimated assuming a circular profile). Experiments were conducted at 22°C using SLControl software (Campbell and Moss, 2003).

### Para-nitroblebbistatin preparation and incubation of samples

Separate sets of solutions with pCa values ranging from 9.0 to 4.5 and pN-Bleb concentrations of 0, 1, 10, or 50 μM were generated. The final percentage of DMSO in every experimental solution was 1.33%. Half of the preparations were used to assess tension-pCa relationships. Each of these preparations was initially tested in control solutions (0 pN-Bleb) with pCa values ranging from 9.0 to 4.5. The preparation was then immersed for 5 min in a pCa 9.0 solution containing 1, 10, or 50 μM pN-Bleb. Additional measurements were then performed using solutions containing the chosen pN-Bleb concentration and pCa values ranging from 9.0 to 4.5. The other half of the preparations were used to assess force-velocity relationships. These samples were only tested in pCa 4.5 solutions with 0 pN-Bleb (control) and then a chosen experimental pN-Bleb concentration. These experimental designs ensured that each preparation could act as its own control and minimized the progressive decline in contractile force (experimental run-down) that occurs when permeabilized preparations are subjected to repeated activations.

### *In situ* mechanical measurements

Multicellular preparations were activated in solutions with pCa values ranging from 9.0 to 4.5. Once tension reached steady-state, the preparations were rapidly shortened by 20%, held for 20 ms, and then re-stretched to their original length. All experiments were performed at a sarcomere length of 2.25 μm. The rate of tension recovery (*k*_tr_) was then calculated by fitting the portion of the force record immediately after the re-stretch with a single exponential function of the form F(t) = A + B (1-exp(-*k*_tr_t)), where F(t) is the force at time t, and A and B are constants.

Ca^2+^ sensitivity (pCa_50_) values were calculated by fitting the steady-state force data to a modified Hill equation of the form F = F_pas_ + F_Ca_ ([Ca^2+^]^n^/([Ca^2+^]^n^ + [Ca^2+^_50_]^n^)). In this equation, F_pas_ is the force measured in pCa 9.0 solution, F_Ca_ is Ca^2+^ activated force, n is the Hill coefficient, and [Ca^2+^_50_]^n^ is the free Ca^2+^ concentration required to develop half the maximum Ca^2+^-dependent force.

To measure shortening velocity and power, the multicellular preparations were allowed to shorten for 80 ms against pre-set loads that ranged from 0 to 100% of the maximum tension measured in pCa 4.5 solution. The shortening velocity in each trial was calculated from the slope of a straight line fitted to a plot of fiber length against time during the final 50 ms of the force clamp. The mean force was also determined during this time. The resulting data were then fitted using a hyperbolic equation of the form (F+a) (V+b) = (F_0_+a) b, where F is the force developed at a shortening velocity of V, F_0_ is the isometric force and a and b are constants with dimensions of force and velocity respectively. V_max_ was determined by extrapolating the force-velocity curve to zero load. Power values (P) were calculated as the product of force and velocity. Power-force curves were calculated by fitting the individual data points with a curve of the form P = F b (((F_0_+a)/(F+a))−1). Maximum power was defined as the maximum value of this curve.

### Statistics for *in situ* muscle mechanics on human samples

Data were analyzed using linear mixed models. These are statistical hypothesis tests that are similar to ANOVA procedures but which allow for the fact that multiple samples were analyzed from each heart (Haynes et al., 2014). This increases the statistical power of the hypothesis test in this type of experimental design. Compound symmetry was assumed for the covariance structure and *post-hoc* analyses were performed using Tukey–Kramer corrections. *P* < 0.05 were considered significant. Data are reported as mean ± SEM.

## Results

We have examined the impact of pN-Bleb on the motor properties of recombinantly expressed human β-cardiac myosin subfragment 1 (M2β-S1) and the force and velocity properties of human myocardium. When possible, we performed the motor function assays and muscle mechanics studies under very similar conditions (temperature and buffer) to allow comparison of the impact of the drug on muscle fiber mechanics and isolated myosin motor performance. We also examined the impact of pN-Bleb on the heavy meromyosin fragment of chicken skeletal muscle myosin (Sk HMM) with *in vitro* motility and actin-activated ATPase assays, which allowed a comparison of the specificity of pN-Bleb for these two myosin isoforms.

### *In vitro* motility of M2β-S1 and Sk HMM

**Figure 3:**
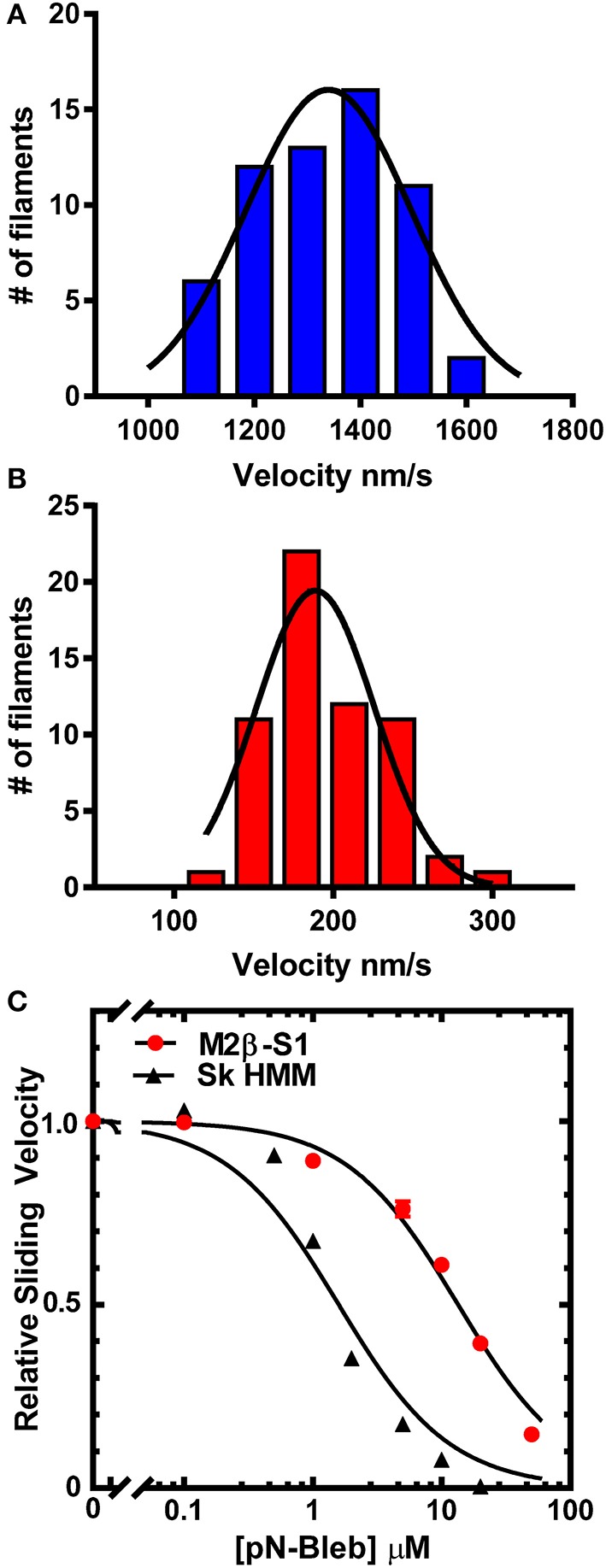
**Impact of pN-Bleb on ***in vitro*** motility**. The sliding velocity in the *in vitro* motility assay was examined with M2β-S1 in the **(A)** absence (1% DMSO) and **(B)** presence of 50 μM pN-Bleb. The average sliding velocity was determined by examining 60 filaments from two different protein preps. The velocities were binned and fit to a Gaussian function to determine the mean ± SEM velocity in the presence and absence of pN-Bleb (195 ± 4.65 and 1334 ± 16.79 nm/s, respectively). **(C)** The average sliding velocities were plotted as a function of pN-Bleb concentration, which allowed determination of the IC50 for M2β-S1 (13.3 ± 0.1 μM) and Sk HMM (1.6 ± 0.2 μM).

The *in vitro* motility assay was utilized to examine the impact of pN-Bleb on the motile properties of purified M2β-S1 and Sk HMM. The sliding velocity produced by M2β-S1 in the *in vitro* motility assay (motility buffer at 22°C) was determined in the presence of varying concentrations of pN-Bleb or 1% DMSO by examining 2 separate protein preparations at a loading concentration of 0.48 μM (Figure 3). Our previous density-dependent *in vitro* motility studies with M2β-S1 demonstrated that this motor density (0.48 μM loading) was saturating (Swenson et al., 2016). The presence of 1% DMSO had a minor impact on *in vitro* motility (the average velocity was 1398 ± 19 and 1261 ± 22 nm/s in the absence and presence of 1% DMSO, respectively). The data from 2 preps was pooled together (60 filaments) to determine the average sliding velocity at each pN-Bleb concentration. There was an 85% inhibition of the sliding velocity in the presence of 50 μM pN-Bleb (Figures 3A,B) and the IC50 (13.3 ± 0.14 μM) was estimated from the concentration dependence (Figure 3C). The *in vitro* motility of Sk HMM was performed in MOPS 20 buffer at 24°C, since it was difficult to obtain results in the higher ionic strength motility buffer that was utilized with M2β-S1. We found that the IC50 for Sk HMM (1.6 ± 0.3 μM) was indicative of a higher specificity of the drug for Sk HMM compared to M2β-S1.

### Actin-activated ATPase activity of M2β-S1 and Sk HMM

**Figure 4:**
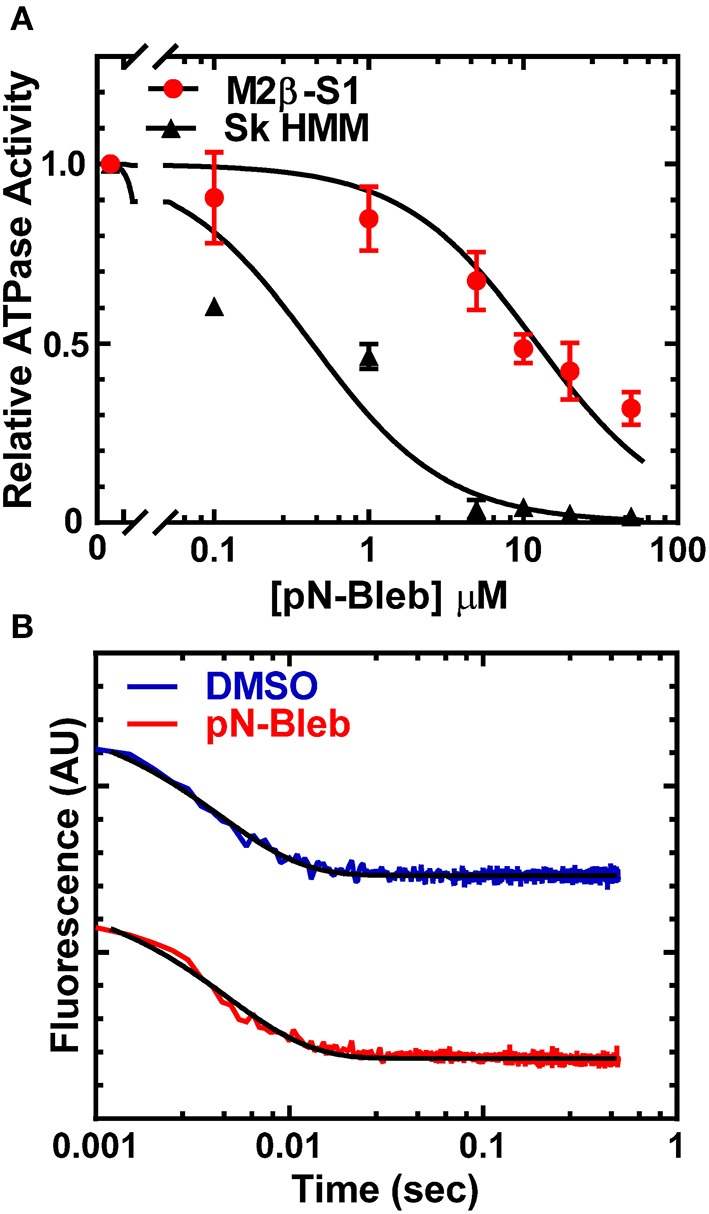
**Impact of pN-Bleb on ATPase Activity and ADP-release**. **(A)** The actin-activated ATPase in the presence of 40 μM actin was determined as a function of pN-Bleb concentration, which allowed determination of the IC50 for M2β-S1 (12.3 ± 1.8 μM) and Sk HMM (0.43 ± 0.11 μM). The data at each pN-Bleb concentration represents the average ± SD from two separate preparations. **(B)** The ADP-release rate constant was determined in the presence and absence of pN-Bleb by mixing a complex of acto-M2β-S1.*mant*ADP with excess ATP and monitoring the mant fluorescence decrease. The fluorescence transients displayed represent the average of 4–5 transients and are fit to a single exponential function.

We examined the impact of pN-Bleb on the actin-activated ATPase of purified M2β-S1 and Sk HMM. We examined the ATPase activity in MOPS 20 buffer, since the higher ionic strength of the motility buffer was not feasible for examining actin-activated ATPase. The ATPase assay with M2β-S1 was performed at 22°C in the presence of 40 μM actin and demonstrated that pN-Bleb inhibits actin-activated ATPase in a dose-dependent manner (Figure 4A). The determined IC50 was similar to that determined in the *in vitro* motility assay (12.3 ± 1.8 μM). We also performed ATPase assay experiments with Sk HMM in similar conditions (MOPS 20 buffer and 25°C) and found the IC50 (0.4 ± 0.1 μM) indicated a higher specificity for Sk HMM compared to M2β-S1.

### ADP release rate constant of M2β-S1

The ADP release rate constant is thought to be an important determinant of the time period that myosin is attached to actin during the ATPase cycle (Siemankowski and White, 1984; Siemankowski et al., 1985). Therefore, we utilized mant labeled ADP to monitor the release of ADP from acto-M2β-S1 in MOPS 20 buffer at 22°C, which was identical to the conditions of the actin-activated ATPase assay. The fluorescence transients were fit to a single exponential function which allowed us to determine the ADP release rate constant (Figure 4B). The results demonstrate that the ADP release rate constant measured with *mant*ADP is very similar in the presence and absence of 50 μM pN-Bleb (208.9 ± 5.1 and 228.6 ± 6.5 sec^−1^, respectively).

### Muscle mechanics of human myocardium

**Figure 5:**
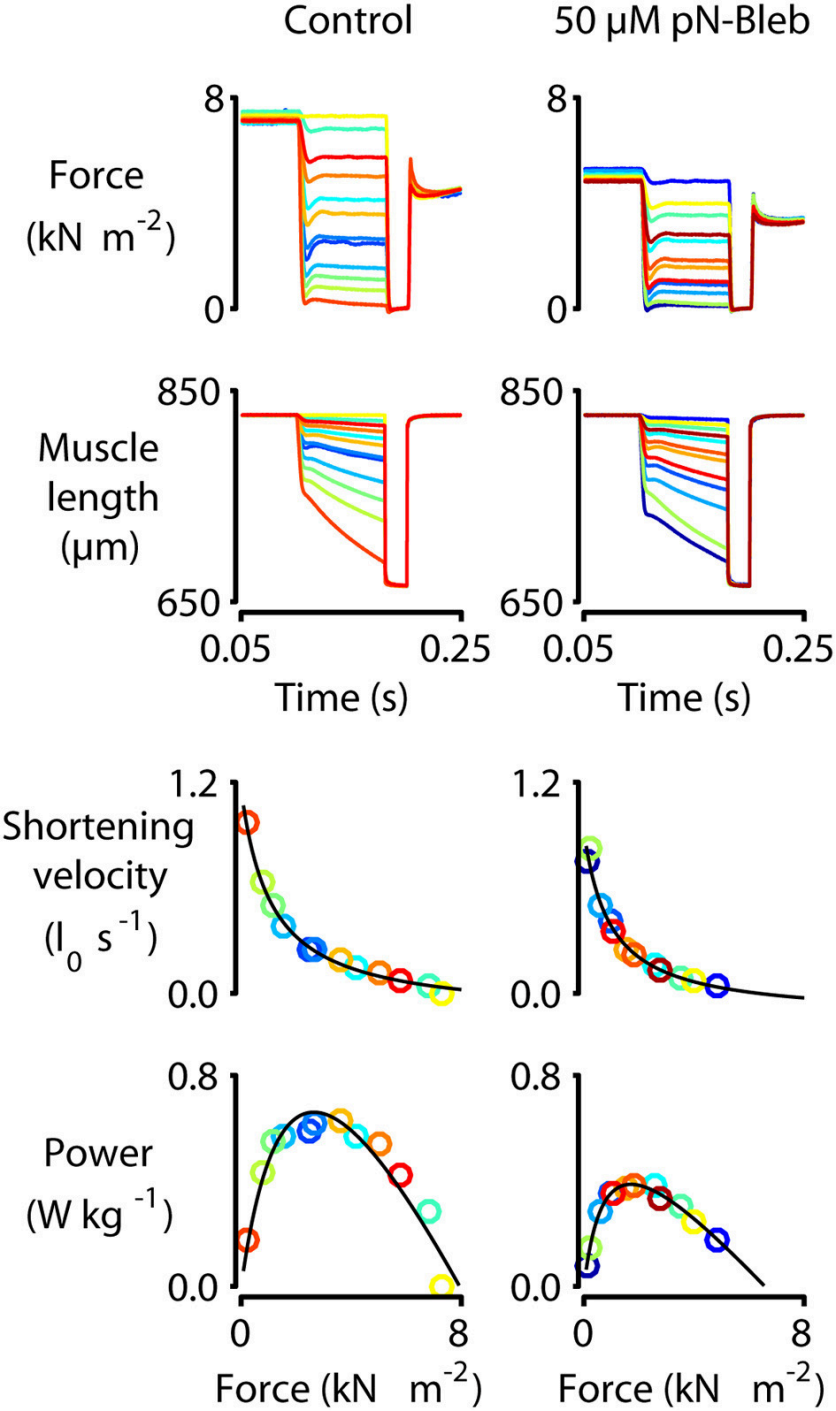
**Force-velocity measurements with human myocardium**. Representative mechanics experiments measuring force-velocity and power. Panels show raw force and raw length traces superposed (top two rows) and force-velocity and force-power curves (bottom two rows) for a single representative preparation measured under control conditions (left column, 0 pN-Bleb) and in the presence of 50 μM pN-Bleb (right column). The symbols showing force, power, and shortening velocity are drawn in the same color as the raw traces from which they were calculated. As described in the Section Methods and by Haynes et al. (2014), these data were obtained by first activating the preparation in pCa 4.5 solution and then allowing it to shorten against loads ranging from 0 to 100% isometric force in successive trials. The shortening velocity was calculated for each trial from the slope of the muscle length against time trace. Similarly, the mean force during shortening was calculated from the force record. Each single trial thus yielded a single data point on the force-velocity plot. Power values were calculated as the product of force and velocity.

**Figure 6:**
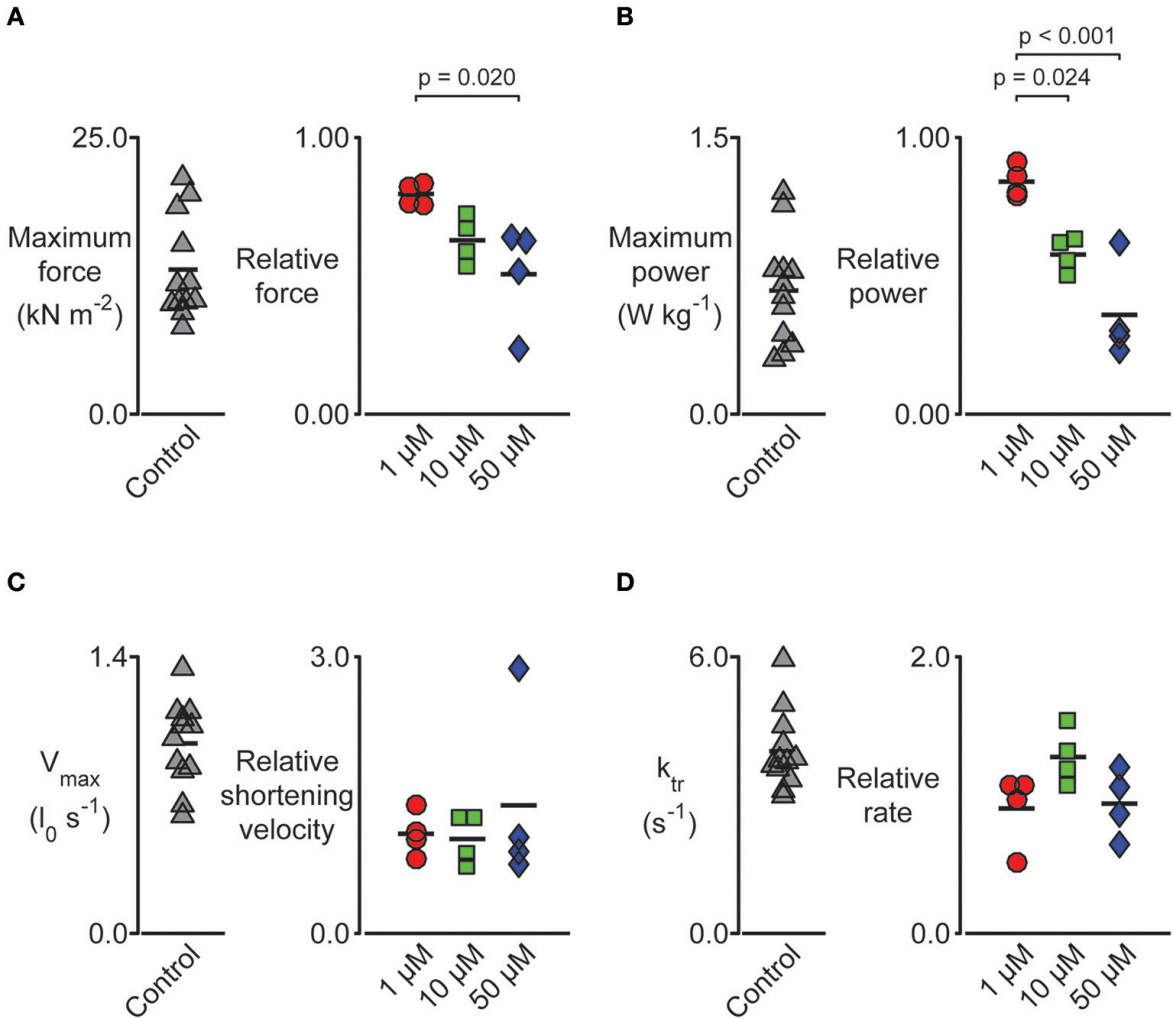
**Impact of pN-Bleb on permeabilized myocardium muscle mechanics**. Left-hand panels show raw data measured in pCa 4.5 solution plus 1.33% DMSO. Right-hand panels show data measured in pCa 4.5 solution plus 1.33% DMSO and either 1, 10, or 50 μM pN-Bleb. These values are normalized to the control (zero pN-Bleb) data measured for that preparation to improve statistical power. *Post-hoc* tests show the results of a linear mixed model statistical analysis as described in the main text. Each point shows data from a single preparation obtained from one of 4 hearts. **(A)** The maximum force was impacted by pN-Bleb in a dose-dependent manner. **(B)** The maximum power was reduced in the presence of pN-Bleb. **(C)** The maximum velocity and **(D)** rate of force development (k_tr_) were unchanged by pN-Bleb.

We performed a series of mechanical tests to determine how pN-Bleb impacted the Ca^2+^-dependence of contractile force and tension-recovery kinetics, and the shortening velocity and power output measured at maximum Ca^2+^activation. Figure 5 shows representative experimental records (top 2 rows) for the force-velocity/force-power measurements and curves calculated from these records (bottom 2 rows; Experimental details are provided in Section Methods and in the Figure legend). These measurements yielded data quantifying isometric force (Figure 6A), maximum power (Figure 6B) and maximum shortening velocity (Figure 6C). Summary data for *k*_tr_, the rate of tension recovery, are shown in Figure 6D. As described in Section Methods, these values were obtained by measuring how quickly force recovered toward steady-state after a large shortening/re-stretch perturbation (raw traces not shown). The statistical hypothesis tests showed that 50 μM pN-Bleb reduced both isometric force (Figure 6A) and maximum power (Figure 6B) by ~50% but did not produce significant changes in either maximum shortening velocity (Figure 6C) or k_tr_ (Figure 6D). Isometric force normalized to cross-sectional area is lower for chemically permeabilized human myocardial samples than it is for some other types of muscle preparations, which we have demonstrated previously (Haynes et al., 2014).

**Figure 7:**
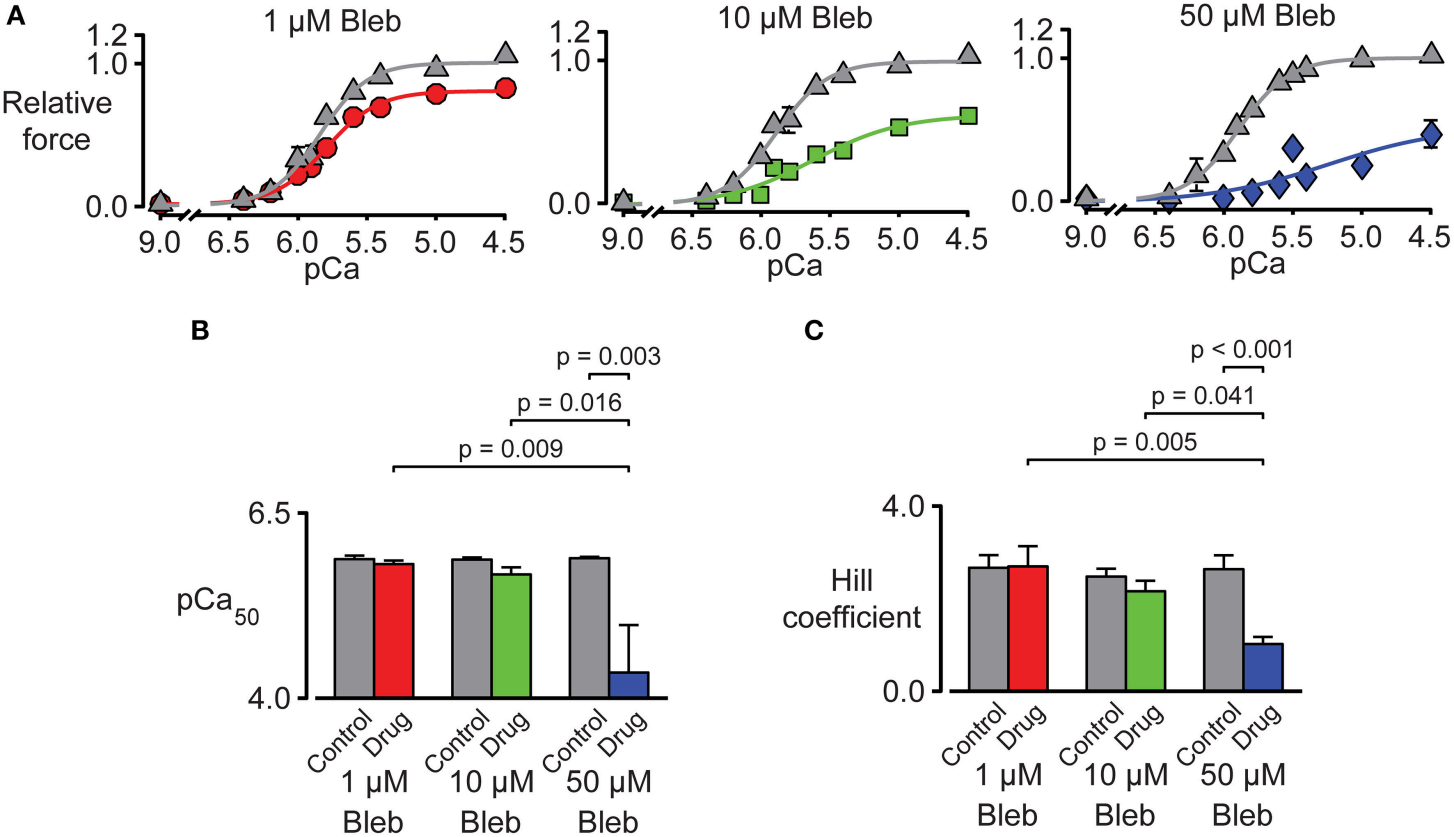
**Impact of pN-Bleb on Ca^**2+**^ sensitivity and isometric force. (A)** Three panels showing tension-pCa plots for samples of chemically permeabilized human myocardium activated in solutions with pCa values ranging from 9.0 to 4.5. The control (gray) data points show force values measured in the presence of 1.33% DMSO. The red, green, and blue data points show force measured in 1.33% DMSO plus 1, 10, or 50 μM pN-Bleb. All force values are normalized to the force measured in pCa 4.5 solution in DMSO with 0 pN-Bleb. Different preparations were used to test each drug concentration. **(B)** The Ca^2+^ sensitivity represented by pCa_50_. Bars show mean ± SEM pCa_50_ values measured in the presence of 1, 10, or 50 μM pN-Bleb. *Post-hoc* tests show the results of a linear mixed model statistical analysis as described in the main text. **(C)** The Hill coefficient results are displayed as in **(B)**.

Tension-pCa curves were generated in additional experiments and are plotted in Figure 7A. As shown during the force-velocity measurements, pN-Bleb reduced isometric force in a dose-dependent manner. pN-Bleb also reduced the Ca^2+^-sensitivity (pCa_50_ values; Figure 7B) and the Hill coefficient (Figure 7C). However, these effects were only significant at the 50 μM concentration which suggests that effects of pN-bleb on Ca^2+^ activation are relatively modest.

## Discussion

Directly targeting human cardiac myosin with small molecule allosteric regulators has been proposed as a therapeutic strategy for several forms of heart failure (Malik et al., 2011; Tardiff et al., 2015; Green et al., 2016). We demonstrate the impact of a myosin inhibitor, which is a modified version of the well-studied Bleb, on human cardiac muscle myosin at the molecular and tissue levels. Although, this drug is not specific for cardiac myosin since it has been demonstrated to inhibit several other muscle and non-muscle myosins, it still serves as a model to examine mechanistically how inhibition of cardiac myosin can be accomplished and how this will impact muscle performance. We find that pN-Bleb reduces the *in vitro* motility of cardiac myosin likely because it decreases the myosin duty ratio by inhibiting the transition into the strongly bound states. In muscle mechanic studies we find that pN-Bleb has no impact on shortening velocity or the rate of force development while the decrease in steady-state force, Ca^2+^ sensitivity, and power are also indicative of a reduced duty ratio.

### The motor properties of M2β in the presence of pN-Bleb

The *in vitro* motility results in the current study demonstrate a pN-Bleb concentration-dependent reduction in sliding velocity. We also observed a similar concentration-dependent reduction in the actin-activated ATPase, which suggests the drug inhibits a similar step in the ATPase cycle in both assays. In light of the previous studies on Bleb, it is likely that pN-Bleb traps cardiac myosin in a weakly bound state that reduces the rate of actin-activated phosphate-release. We find that pN-Bleb does not alter the ADP-release rate constant which typically correlates with the time myosin is attached to actin and is an important determinant of maximum velocity and duty ratio. Thus, pN-Bleb acts by stabilizing the weakly bound conformation and in the *in vitro* motility assay the reduction in myosin heads that productively attach to actin and produce force creates a situation similar to what is observed at low motor densities. When the number of force generating heads in the motility assay decreases, it is proposed that the period of time between myosin attachments becomes rate-limiting (Uyeda et al., 1990; Harris and Warshaw, 1993). Interestingly, we did not observe a reduction in shortening velocity in the human cardiac muscle mechanics studies. These results may reflect the structural organization in muscle which has many myosin heads in close proximity to the actin thin filament and thus is not as sensitive to this type of inhibition. The original theory of muscle contraction outlined by Huxley (1957) proposed that unloaded shortening velocity was independent of the number of cycling myosin crossbridges. Furthermore, it has been demonstrated that only 1–4 myosins per thick filament are required to sustain maximum velocity (Fusi et al., 2016). The reduction in steady-state force and power is consistent with the proposed mechanism of reducing the myosin duty ratio and therefore the number of myosin heads available to generate force. Thus, the mechanism of inhibition utilized by this drug is advantageous because at moderate doses it does not change the kinetics of contraction while it does effectively reduce steady-state force and power. In patients that are hyper-contractile this mechanism may work well since it could normalize the force velocity relationship and power without altering the systolic contraction time and relaxation kinetics. It is also important to consider the impact of this type of inhibition on shortening velocity in the presence of load since this is the more physiologically relevant situation in the heart. From the force-velocity experiments (Figure 5) it is clear that pN-Bleb alters shortening velocity in the presence of load and thus the systolic contraction time could be impacted. We did not directly measure the impact of pN-Bleb on the phosphate release rate constant and this measurement as well a detailed examination of all of the transient kinetics steps in the M2β-S1 ATPase cycle will be important to examine in future studies.

Interestingly, the specificity of pN-Bleb for skeletal muscle myosin was nearly 10-fold higher than human cardiac myosin based on the measured IC50 in the motility assay and 30-fold higher based on ATPase assays. The ATPase IC50 value we determined for skeletal muscle myosin was similar to that reported in the literature (Képiró et al., 2014). These results demonstrate that the binding affinity of pN-Bleb for cardiac myosin may be weaker than skeletal myosin or that the structural state that favors pN-Bleb binding is more significantly populated in skeletal. Limouze et al. (2004) determined the specificity of Bleb for many different muscle and non-muscle myosins and found considerable variability. Further high resolution structural studies are necessary to evaluate the structural details of the Bleb binding pocket which may be a useful site for rationally designing myosin inhibitors.

### Impact of pN-Bleb on human myocardium

The muscle mechanics data clearly demonstrate that pN-Bleb reduced isometric force in a dose-dependent manner. However, the effects of pN-Bleb on Ca^2+^ sensitivity need to be interpreted with care. Although, the pCa_50_ values and Hill coefficients were significantly reduced by a pN–Bleb concentration of 50 μM, the lower concentrations of pN-Bleb did not produce marked effects. It's also unclear whether isometric force was completely saturated in a pCa 4.5 solution in the presence of 50 μM pN-Bleb (note that the tension-pCa curve did not reach a flat plateau in Figure 7A). These data could indicate that a high concentration of pN-Bleb desensitizes the thin filaments by reducing the myosin duty ratio. However, the low Hill coefficients and decreased pCa_50_ values measured in the presence of 50 μM pN-Bleb could also be explained by a progressive reduction in force development during the experiments. Dou et al. (2007) showed that the effects of Bleb on force development in mouse papillary muscle and trabeculae were time-sensitive and that force took almost 30 min to stabilize after application of the drug. Similar time-dependent effects in human myocardium might produce tension-pCa data similar to those shown in Figure 7A.

### Considerations of thin filament regulation

Our results are consistent with the hypothesis that there is a correlation between the myosin duty ratio and Ca^2+^ sensitivity and that this relationship can be tuned with small molecule drugs that alter the myosin duty ratio. It is well established that strong-binding cross-bridges have a positive feedback regulation on thin filament Ca^2+^ sensitivity, which stabilizes the Ca^2+^ bound state of troponin C (Kobayashi et al., 2008). Therefore, changes in the number of strong binding cross-bridges on thin filaments, determined by duty ratio, could impact the thin filament Ca^2+^ sensitivity. Thus, changes in duty ratio could explain the disrupted myofilament Ca^2+^ sensitivity observed in studies of M2β mutations. Mutations in M2β can increase or decrease duty ratio (proposed to occur in HCM and DCM, respectively) by altering the kinetics of individual steps in the ATPase cycle and perturbing isometric force generation and thin filament Ca^2+^ sensitivity. While it seems clear that HCM and DCM mutations in tropomyosin and the troponin complex are Ca^2+^ sensitizing and desensitizing, respectively (Sommese et al., 2013; Spudich et al., 2016), it remains controversial how M2β cardiomyopathy mutations impact thin filament Ca^2+^ sensitivity. The reduced Ca^2+^ sensitivity and Hill coefficient of the human myocardial samples was observed in the presence of pN-Bleb, but the difference is only significant at 50 μM concentration. Therefore, our results suggest the strategy of altering the Ca^2+^ sensitivity by altering the myosin duty ratio may be feasible.

### Considerations of thick filament regulation

Regulation at the level of the thick filament can occur by formation of the SRX and drugs that alter the stabilization of this state could be utilized to enhance or depress force generation in cardiac muscle. The SRX has been identified as a state in striated muscle in which myosin heads are folded back on the backbone of the myosin thick filament (Hooijman et al., 2011). In addition, cryo-EM (Wendt et al., 2001; Craig and Woodhead, 2006; Zoghbi et al., 2008), and X-ray diffraction (Linari et al., 2015) studies have also demonstrated the presence of the folded back state of myosin in various muscle types from different species. The SRX provides a protective mechanism for maintaining a pool of quiescent myosin heads that have slow ATP hydrolysis (Fusi et al., 2015). Regulatory light chain phosphorylation, ablation of myosin binding protein C, and mechanical stress may impede the formation of the SRX (Linari et al., 2015; Kampourakis et al., 2016; McNamara et al., 2016). It has been proposed that cardiomyopathy associated mutations in myosin and thick filament associated proteins could disrupt contractile properties by altering the formation of the SRX, which ultimately impacts the number of cross-bridges capable of generating force (Kampourakis et al., 2016). Since the previous work on the parent drug suggests that Bleb may stabilize the SRX (Zhao et al., 2008; Xu et al., 2009; Wilson et al., 2014), the decrease in steady-state force and Ca^2+^ sensitivity in the presence of pN-Bleb in the current study could be at least partially attributed to stabilization of the SRX.

### Comparison to other small molecule regulators

Currently, other drugs are being pursued that directly enhance or depress the activity of human M2β. Omecamtiv Mecarbil (OM) is a cardiac myosin allosteric modulator that is currently in Phase II clinical trials to treat systolic heart failure (Cleland et al., 2011; Greenberg et al., 2015; Teerlink et al., 2016). OM is specific to cardiac myosin with no effect on smooth or skeletal muscle myosin (Malik et al., 2011). While many studies have been done to investigate the impact of OM on muscle fibers in different animal models, the molecular mechanisms of how OM impacts cardiac myosin still remain unclear (Malik et al., 2011; Mamidi et al., 2015; Nagy et al., 2015; Utter et al., 2015). Steady state and transient kinetics have been examined to investigate the detailed mechanism of the impact of OM on purified porcine cardiac myosin (Liu et al., 2015). The kinetic analysis demonstrated that OM shifts the ATP hydrolysis equilibrium constant toward products and favors phosphate release, while the ADP release rate constant is unchanged. These changes translate to an increase in the number of force-generating cross-bridges bound to the thin filament in the presence of OM (Liu et al., 2015), which is consistent with the enhanced force production observed in muscle mechanic studies. The increase in the number of strongly bound force-generating heads in the presence of OM may exert an internal drag on the thin filaments that decreases sliding velocity. The internal drag could also slow the ADP release rate constant by a strain-dependent mechanism which would slow the detachment rate and thus the sliding velocity. Consistent with this hypothesis, the presence of OM dramatically inhibited the sliding velocity of porcine HMM (15- to 20-fold decrease) as measured in the *in vitro* motility assay in several studies (Wang et al., 2014; Liu et al., 2015; Winkelmann et al., 2015). OM has been found to slow force development as well as activation and relaxation kinetics but increase myofilament Ca^2+^ sensitivity in isolated cardiomyocytes from rodent models (Mamidi et al., 2015; Nagy et al., 2015; Utter et al., 2015). Further study is necessary to determine the mechanism of how OM slows filament sliding in muscle mechanics and *in vitro* motility studies as well as how the impact on motor properties influences the contractile performance of the heart.

Another recent study identified a novel cardiac myosin inhibitor, MYK-461, which is proposed to suppress cardiac myosin motor function by decreasing duty ratio. MYK-461 reduces the overall ATPase activity of cardiac myosin in a dose dependent-manner (with a 90% maximal inhibition; Green et al., 2016). Transient kinetic experiments suggested that MYK-461 slows down the phosphate release step without changing the ADP release rate constant. Myofibril studies showed that the presence of 1 μM MYK-461 reduced maximal tension by 70%. Oral administration of MYK-461 decreased fractional cardiomyocyte shortening in wild-type and HCM-mutant mice, but importantly prevented the development of an HCM phenotype in the mutant mice. Therefore, MYK-461 normalizes the hyper-contractile properties of cardiac muscle by decreasing the power output, and suppresses the development of ventricular hypertrophy in mice carrying heterozygous human mutations (R403Q, R453C, R719W) in M2β (Green et al., 2016).

## Conclusions

We find that a M2β-S1 inhibitor (pN-Bleb) that acts by reducing strong actin binding without altering detachment kinetics may be advantageous for reducing the myosin duty ratio. The impact of the drug on muscle fiber studies demonstrates that this type of inhibition reduces steady-state force, power, and Ca^2+^ sensitivity which may help treat hyper-contractile patients. The maximum shortening velocity is not very sensitive to this type of inhibition in a muscle fiber while it is quite sensitive when examined in the motility assay with purified M2β-S1. Thus, it is important to consider the unique structural organization of muscle and how this may cause differences when comparing the *in vitro* motility and muscle mechanic studies. The ability to develop motility assays that better mimic a muscle fiber and retain the structural organization of the thick and thin filaments as well as the key regulatory proteins will be extremely helpful in future studies. The exciting new drug, MYK-461, which appears to act in a similar fashion to pN-Bleb, was successfully used to treat HCM in a mouse model. Further studies are necessary to determine if treatment of HCM with drugs that reduce the myosin duty ratio will be successful for a variety of HCM mutations. In addition, it will be interesting to determine if drugs that increase the myosin duty ratio, such as OM, can be used to treat patients with mutations that cause hypo-contractility.

## Ethics statement

Study involved animal subjects; Approved by University of Kentucky Institutional Review Board, protocol 08-0338; Patients gave informed consent for sample donation before undergoing cardiac surgeries. Only specimens that were removed as part of normal clinical care and that would otherwise have been discarded were used in this study. No vulnerable populations.

## Author contributions

CY and KC designed research; AM-C provided critical reagents; WT, CB, SW, and CY performed research; WT, CB, KC, and CY analyzed the data; WT, CB, KC, and CY wrote paper. All authors approved the final version of the manuscript.

### Conflict of interest statement

The authors declare that the research was conducted in the absence of any commercial or financial relationships that could be construed as a potential conflict of interest.
